# The *in situ* Production of Aquatic Fluorescent Organic Matter in a Simulated Freshwater Laboratory Model

**DOI:** 10.3389/fmicb.2022.817976

**Published:** 2022-02-24

**Authors:** Eva M. Perrin, Robin M. S. Thorn, Stephanie L. Sargeant, John W. Attridge, Darren M. Reynolds

**Affiliations:** ^1^Centre for Research in Biosciences, University of the West of England, Bristol, United Kingdom; ^2^Chelsea Technologies Ltd., East Molesey, United Kingdom

**Keywords:** dissolved organic matter, freshwater, microbial processing, nutrient loading, fluorescence

## Abstract

Dissolved organic matter (DOM) is ubiquitous throughout aquatic systems. Fluorescence techniques can be used to characterize the fluorescing proportion of DOM, aquatic fluorescent organic matter (AFOM). AFOM is conventionally named in association with specific fluorescence “peaks,” which fluoresce in similar optical regions as microbially-derived proteinaceous material (Peak T), and terrestrially-derived humic-like compounds (Peaks C/C+), with Peak T previously being investigated as a tool for bacterial enumeration within freshwaters. The impact of anthropogenic nutrient loading on the processing of DOM by microbial communities is largely unknown. Previous laboratory studies utilizing environmental freshwater have employed growth media with complex background fluorescence, or very high nutrient concentrations, preventing the investigation of AFOM production under a range of more representative nutrient concentrations within a matrix exhibiting very low background fluorescence. We describe a laboratory-based model with *Pseudomonas aeruginosa* that incorporates a low fluorescence growth matrix consisting of a simulated freshwater (SFW), representative of low-hardness freshwater systems allowing controlled nutrient conditions to be studied. The effects of microbial processing of DOM as a function of available nitrogen, phosphorous, and dissolved organic carbon (DOC) in the form of glucose were investigated over 48 h at highly resolved time increments. The model system demonstrates the production of a range of complex AFOM peaks in the presence and absence of DOC, revealing no linear relationship between cell numbers and any of the peaks for the bacterial species studied, with AFOM peaks increasing with microbial cell number, ranging from 55.2 quinine sulfate units (QSU) per 10^6^ cells to 155 QSU per 10^6^ cells (*p* < 0.05) for Peak T during the exponential growth phase of *P. aeruginosa* under high nutrient conditions with 5 mg L^−1^ DOC. Nutrient and DOC concentration was found to cause differential production of autochthonous- or allochthonous-like AFOM, with lower DOC concentrations resulting in higher Peak T production relative to Peaks C/C+ upon the addition of nutrients, and high DOC concentrations resulting in higher Peak C/C+ production relative to Peak T. Our results show the production of allochthonous-like AFOM from a simple and non-fluorescent carbon source, and provide uncertainty in the use of Peak T as a reliable surrogate for specific bacterial enumeration, particularly in dynamic or nutrient-impacted environments, pointing toward the use of fluorescence as an indicator for microbial metabolism.

## Introduction

Dissolved organic matter (DOM) is one of the largest reservoirs of carbon on the planet, representing a source of both fixed and bioavailable carbon that is ubiquitous throughout aquatic environments (Hedges, 1992; Cole et al., 2007; Trimmer et al., 2012). In freshwater systems, DOM is a largely heterogeneous mixture of organic compounds, the type and quantity of which is representative of surrounding catchment characteristics, as well as being influenced by processes such as photodegradation and *in situ* microbial processing. DOM is conventionally divided into two categories; allochthonous material, which represents carbon that has been transported into the fluvial system from surrounding terrestrial environments, is considered to represent complex, high molecular-weight humic and fulvic organic carbon, and autochthonous material represents carbon that has been generated within the fluvial system by microbial processes is regarded as simpler, proteinaceous, and lower molecular-weight in nature (McKnight et al., 2001; Leenheer and Croué, 2003 Baker and Spencer, 2004; Hudson et al., 2007).

A fraction of DOM in aquatic environments exhibits fluorescence properties, and this is known as aquatic fluorescent organic matter (AFOM). AFOM can be analyzed using fluorescence spectroscopy, providing a rapid and sensitive means of investigating the processing and transport of carbon in aquatic systems. AFOM is classified using specific peak nomenclature (Coble et al., 2014), based on the presence of observed fluorescence peaks which appear within optical regions associated with known organic compounds. AFOM associated with microbially-derived compounds is known as “autochthonous.” Peaks within this region have been named Peaks T (λ*_ex_*/λ*_em_* 275/340) and B (λ*_ex_*/λ*_em_* 275/305), which fluoresce in the same optical regions as tryptophan and tyrosine, essential amino acids. This is also known as “protein-like” AFOM. AFOM associated with terrestrially-derived compounds is known as “allochthonous,” and includes peaks known as Peak C (λ*_ex_*/λ_em_ 320–365/420–470), Peak C+ (λ*_ex_*/λ*_em_* 385–420/470–505), and Peak M (λ*_ex_*/λ*_em_* 290–310/370–420). Despite this binary classification, some peaks have been reported to occur as a result of both autochthonous and allochthonous processes, such as Peak M in marine environments (Milbrandt et al., 2010).

Much of the work on AFOM in freshwater systems has focused on utilizing Peak T fluorescence as a tool for microbial enumeration, derived from reported relationships between Peak T fluorescence intensities and primary productivity (Hudson et al., 2007, 2008) or microbial enumeration (Sorensen et al., 2015, 2018). Previous studies have sought to investigate the origins of AFOM and its relationship with microbial processing in marine systems (Yamashita and Tanoue, 2004; Yamashita et al., 2008; Shimotori et al., 2010; Kinsey et al., 2018) and, more recently, in surface waters such as lakes (Guillemette and del Giorgio, 2012; Berggren et al., 2020). These have suggested that AFOM in the humic-like optical region can be produced as a direct result of aquatic microbial activity. Recent studies (Fox et al., 2017, 2018) employing laboratory-based model systems to investigate the origin of freshwater AFOM produced by bacteria *in situ* have provided further evidence to support this. Furthermore, this work has suggested that Peak T fluorescence is associated with microbial metabolism and activity, and is not a reliable indicator for specific cell enumeration as has been previously reported.

Despite recent advances, there is still a dearth of knowledge surrounding the role played by freshwater microbes within *in situ* AFOM processing (Anderson et al., 2019). Inland freshwaters are known to play a disproportionately large role in global carbon cycling, despite covering less than 4% of the Earth’s surface (Cole et al., 2007; Downing, 2008; Tranvik et al., 2009). Furthermore, the inherent complexity of microbial AFOM processing is compounded by anthropogenically-induced perturbations, particularly agricultural activities, which often lead to nutrient loading as a result of runoff from cultivated land surrounding a river catchment. Over recent decades, the availability of nutrients such as reactive nitrogen and phosphorus species has greatly increased in surface waters, with 38% of European freshwaters impacted by non-point source pollution such as agricultural land-use (Woodward et al., 2012; European Environment Agency, 2018). Recent field-based studies have observed increases in ecosystem respiration and ultimately carbon loss within surface waters as a result of nutrient loading (Kominoski et al., 2018; Manning et al., 2018). There is, however, a need to develop laboratory models to support these field-based studies. Previous approaches for characterizing microbial AFOM production within environmental water matrices (Guillemette and del Giorgio, 2012; Fox et al., 2018) are limited by complex background fluorescence signatures, or the utilization of high-nutrient growth media which do not represent nutrient concentrations observed in the field (Fox et al., 2017, 2018). Therefore, existing models are of limited use for investigating the effects of controlled nutrient loading on microbial AFOM production.

The work undertaken here seeks to develop and utilize a laboratory-based model for the investigation of nutrient loading on *in situ* bacterial AFOM processing within fresh water systems. A simulated freshwater (SFW) matrix, adapted from Smith et al. (2002), was developed, standardized, and used throughout the study. In contrast to previous laboratory-based studies (Fox et al., 2017, 2018, 2021), the SFW matrices contains concentrations of ionic constituents found within oligotrophic fresh waters (Smith et al., 2002), i.e., contains a low baseline concentration of nitrate, phosphate, and dissolved organic carbon (DOC) in the form of glucose. Importantly, the developed SFW exhibited low background fluorescence, unlike many studies which utilize environmental freshwaters (Fox et al., 2018; Kominoski et al., 2018; Berggren et al., 2020). The main aim of this study was to observe the origin of AFOM *via* bacterial production and processing as a function of nutrient availability using a single species freshwater model system.

## Materials and Methods

### Simulated Freshwater Media

Simulated freshwater was developed using an adapted method from Smith et al. (2002) and is detailed in Table 1. The SFW contains low concentrations of nitrate and phosphate (0 mg L^−1^ P0_4_^3−^ and 0.3 mg L^−1^ NO_3_^−^) and no addition of glucose-DOC. By starting with a baseline low concentration of these nutrients in the initial SFW, subsequent higher quantities of nitrates, phosphates, and DOC can be introduced in their desired concentration. This facilitates the investigation of both low and high nutrient conditions, unlike previous studies which have used growth media with very high baseline nutrient concentrations. All SFW prepared using this method was filter-sterilized using a 0.2 μm cellulose filter (Sartorius Stedim Biotech, Germany) prior to use and all glassware sterilized by autoclaving at 121°C for 15 min. Table 1 outlines the concentrations of all ions present within the SFW. Growth curve experimental conditions are detailed in the section “Bacterial Growth Curve Experimental Conditions.”

**Table 1:** Anion and cation concentrations within the SFW, adapted from Smith et al. (2002).

Chemical constituents	Final ion concentrations (mg L^−1^)	
	Anion	Cation
MgCl_2_	1.458	4.254
CaCl_2_	3.209	5.672
Ca(NO_3_)_2_	0.601	1.860
CaCO_3_	6.814	10.201
Na_2_SO_4_	5.288	11.046
KHCO_3_	0.977	1.525
NaHCO_3_	0.458	1.220

### Nitrate, Phosphate, and DOC Conditions

Concentrations of nitrate (NO_3_^−^) and phosphate (PO_4_^3−^) were added to the SFW matrix at concentrations of 50 and 0.1 mg L^−1^, respectively. Stock solutions of nitrate and phosphate were prepared by the dissolution of sodium nitrate (NaNO_3_^−^) and dipotassium hydrogen orthophosphate (KH_2_PO_4_^−^), respectively in deionized water and added to the SFW prior to bacterial inoculation. The chosen concentrations for nitrate and phosphate were informed by the EU Nitrates Directive and its constituting Water Framework Directive (Directive 2000/60/EC of the European Parliament and of the Council of 23 October 2000 establishing a framework for community action in the field of water policy, 2000), whereby these concentrations are deemed to be high and of concern.

Dissolved organic carbon in the form of glucose was dosed into the model system to investigate bacterial production of AFOM from a simple, bioavailable carbon source. SFW solutions containing 0, 5, and 800 mg L^−1^ of DOC were prepared and added to the SFW prior to bacterial inoculation. A concentration of 5 mg L^−1^ of DOC represents DOC availability in many fluvial systems studied previously (Gao et al., 2017; Noacco et al., 2017) where carbon transport in rivers has been modeled. High, or excess, DOC conditions (800 mg L^−1^ glucose) were also studied. DOC levels this high, although not representative of natural systems, ensured that DOC was not a limiting factor when investigating bacterial AFOM production. This is comparable to previous laboratory-based studies that have used high-nutrient growth media with DOC concentrations in excess (Fox et al., 2017). In addition, 0 mg L^−1^ DOC was used to investigate bacterial AFOM production under conditions with no additional DOC source, therefore a total of three DOC conditions were investigated. The six experimental conditions investigated are outlined in Table 2, with the associated nomenclature used to refer to these conditions during the study.

**Table 2:** Nitrate, phosphate, and dissolved organic carbon (DOC) experimental conditions and their associated nomenclature.

	DOC concentrations		
	0 mg L^−1^ DOC	5 mg L^−1^ DOC	800 mg L^−1^ DOC
0 mg L^−1^ PO_4_^3−^	*SFW0* Low nutrient, no DOC	*SFW2* Low nutrient, limited DOC	*SFW4* Low nutrient, excess DOC
0.3 mg L^−1^ NO_3_^−^			
0.1 mg L^−1^ PO_4_^3−^	*SFW1* High nutrient, no DOC	*SFW3* High nutrient, limited DOC	*SFW5* High nutrient, excess DOC
50 mg L^−1^ NO_3_^−^			

Six conditions of varying nutrients were investigated, with both oligotrophic and high-nutrient conditions observed with three different DOC conditions for each.

### Inoculum Preparation

*Pseudomonas aeruginosa* (NCIMB 8295) was selected for study within the model system due to the ubiquitous nature of this species in the freshwater environment (Sigee, 2004; Elliott et al., 2006; Fox et al., 2017). For culture preparation, 10 ml of nutrient broth was inoculated with a single colony derived from a fresh plate culture. Cultures were incubated for 18–24 h at 37°C, shaking at 120 rotations per minute (RPM). Prior to inoculation, bacterial cells were washed three times in SFW by centrifuging at 13,000 ×× *g* for 3 min to form a pellet. The supernatant was then removed and the pellet resuspended in sterile SFW to remove any residual nutrients. Bacterial cell densities (CFU ml^−1^) were standardized using optical density measurements at 620 nm.

### Bacterial Growth Curve Experimental Conditions

Using a glass conical flask (500 ml), sterile SFW (250 ml) was inoculated with *P. aeruginosa* to a density of 10^5^ CFU ml^−1^, this bacterial density has been previously reported to be representative of freshwater systems (Sigee, 2004). Flasks were incubated at 37°C shaking at 120 RPM under ambient light conditions, and aliquots (10 ml) were extracted for analysis at hourly time points for the first 8 h, and then at 12, 16, 20, 24, 36, and 48 h thereafter. The experimental duration of 48 h at 37°C enabled the observation of AFOM production over three phases of bacterial growth (lag, exponential, and stationary). While it is acknowledged that this temperature does not represent environmental conditions, this 37°C was chosen as it has been found to be the optimum growth temperature for *P. aeruginosa* (Tsuji et al., 1982). As such, this facilitated initiating the bacterial AFOM production with this model system, allowing the investigation of a range of metabolic states over a short period of time. Flasks were vented to maintain gas exchange with the environment, thus the experiment was conducted under aerobic conditions. Sample aliquots were serially diluted in sterile phosphate-buffered saline (Oxoid Ltd., United Kingdom) and plated onto nutrient agar *via* spiral plating (Don Whitley Scientific Ltd., England). Plates were incubated at 37°C for 24 h and viable colonies counted to calculate the CFU ml^−1^. Each experimental condition was repeated on three independent occasions (except for SFW5, which was repeated on seven independent occasions due to high variability) using separate overnight bacterial cultures. All fluorescence and bacterial enumeration measurements were performed in triplicate at each time point.

### Fluorescence Measurements

Inoculated SFW (3 ml) was collected for fluorescence excitation-emission matrices (EEMs) at each time point using an Aqualog® (Horiba Ltd., Japan). The following scan parameters were employed: excitation wavelengths from 200 to 600 nm in 1 nm steps, and emission wavelengths from 247.88 to 829.85 nm in 1.16 nm steps and an integration time of 0.5 s. A 3 ml microquartz cuvette with a 10 mm pathlength was used throughout. Spectra were blank-subtracted using the fluorescent spectra of a fresh SFW sample. EEMs of filter-sterilized SFW were collected to determine whether any background fluorescence was present prior to inoculation of the experiment. In addition, control experiments were undertaken alongside this study with sterile SFW under all six nutrient conditions. From this, the SFW media were found to be non-fluorescent, with <5 QSU present after 48 h under any experimental conditions. Inoculated SFW at 0 h of the experiment showed fluorescence intensities of 2.2 QSU (±2) for Peak T, 0.9 QSU (±0.8) for Peak C, and 0.6 QSU (±0.5) for Peak C+.

### Fluorescence Data Analysis

Raw fluorescence data were processed through a custom Python™ script (Python Software Foundation) to normalize data to QSU and generate EEM maps. The data window was cropped to λ_ex_ 240–490 nm and λ_em_ 250–500 nm, to discount fluorescence data within the deep-UV region which is heavily influenced by instrument noise. This allows for the analysis of data within the UV–visible area, the optical region associated with FDOM peaks of interest. PARAFAC was attempted within this study, however, the simple nature of the growth matrix and single-species biological culture limited its application. As such, no robust model (CORCONDIA >90%) was identified which could adequately explain the dataset. This is likely due to the dominance of the main fluorescence peaks, Peaks T and C+. Instead, peak-picking, an established method (Asmala et al., 2016) used for identifying specific fluorescence peaks, was undertaken. For this, cross-sections of emission values at a set excitation wavelength were obtained using the custom Python™ script, and the mean fluorescence intensity of the peaks was calculated. Peak regions were: Peak T (λ*_ex_*/λ*_em_* 275/340), Peak C (λ*_ex_*/λ*_em_* 340/420–470), and Peak C+ (λ*_ex_*/λ*_em_* 400/470–505). Collected absorbance and transmittance data were used to correct spectra for inner-filter effects (Aqualog®) for both excitation and emission wavelengths, and first and second order Rayleigh Scattering was masked. All fluorescence data are reported in quinine sulfate units (QSU), acquired by normalizing data to the fluorescence generated from 1 μg L^−1^ quinine sulfate at λ_ex_ = 347.5 nm and λ_em_ = 450 nm (Kramer and Herndl, 2004; Mostofa et al., 2013). This allows for standardized quantitative analysis that is comparable within and between studies.

### Statistical Analysis

Statistical analysis of all data was conducted using GraphPad Prism version 9.2.0 for Windows (GraphPad Software, San Diego, CA, United States). A time-series analysis of all time points was undertaken using mixed-effects modeling whereby the significance of the fixed effect was assessed using ANOVA (*p* < 0.05 regarded as significant). This enabled statistical comparison of fluorescence intensity between peaks and between nutrient and DOC conditions over time.

## Results

### AFOM Processing Over 48 h

**Figure 1:**
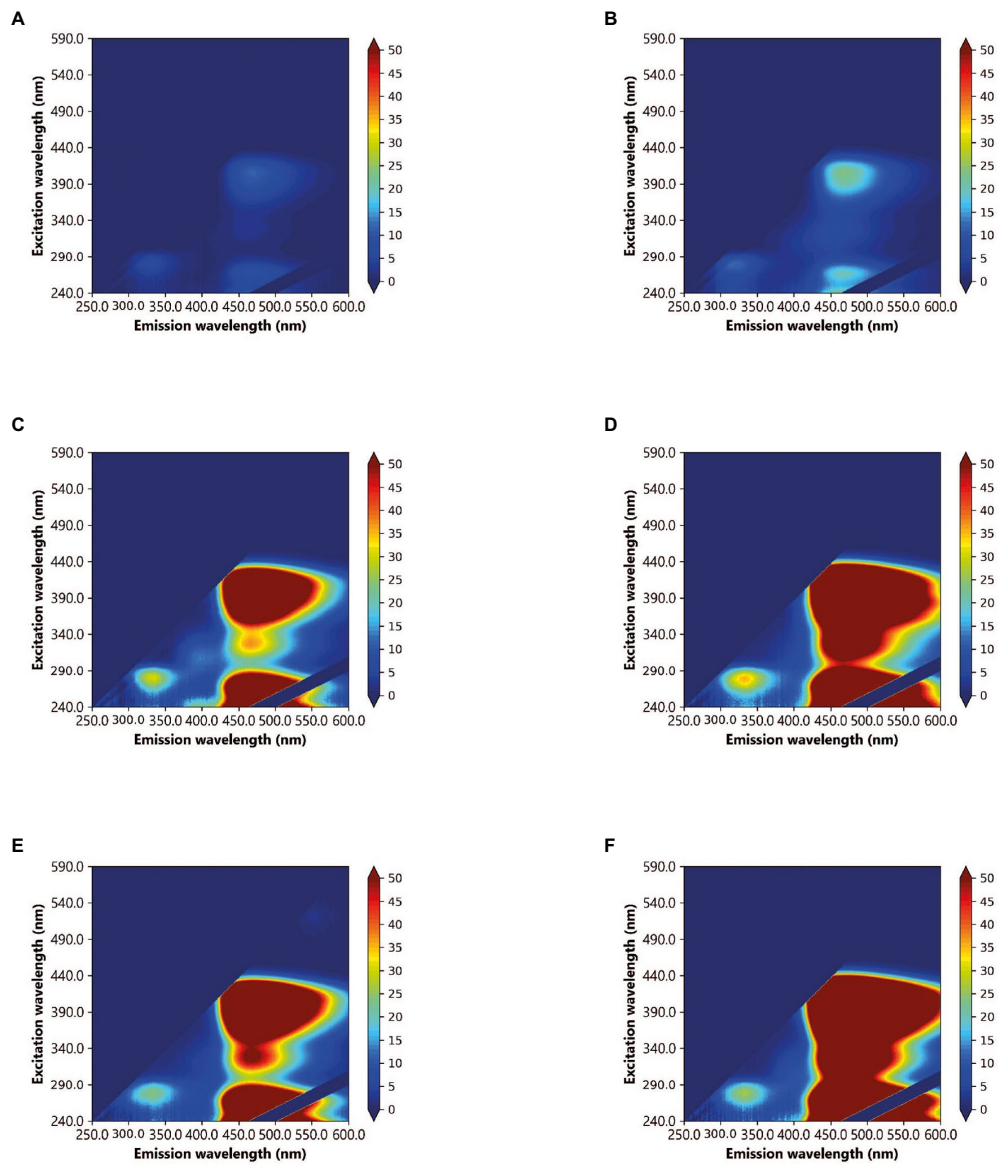
Excitation-emission matrices (EEMs) of *Pseudomonas aeruginosa* monoculture in simulated freshwater (SFW) after a 48 h incubation period, under the following experimental conditions: **(A)** oligotrophic, no carbon (SFW0), **(B)** high nutrient, no carbon (SFW1), **(C)** oligotrophic, limited carbon (SFW2), **(D)** high nutrient, limited carbon (SFW3), **(E)** oligotrophic, excess carbon (SFW4), and **(F)** high nutrient, excess carbon (SFW5). Color bars are displayed in quinine sulfate units (QSU), where 1 QSU = 1 μg L^−1^ quinine sulfate solution.

**Figure 2:**
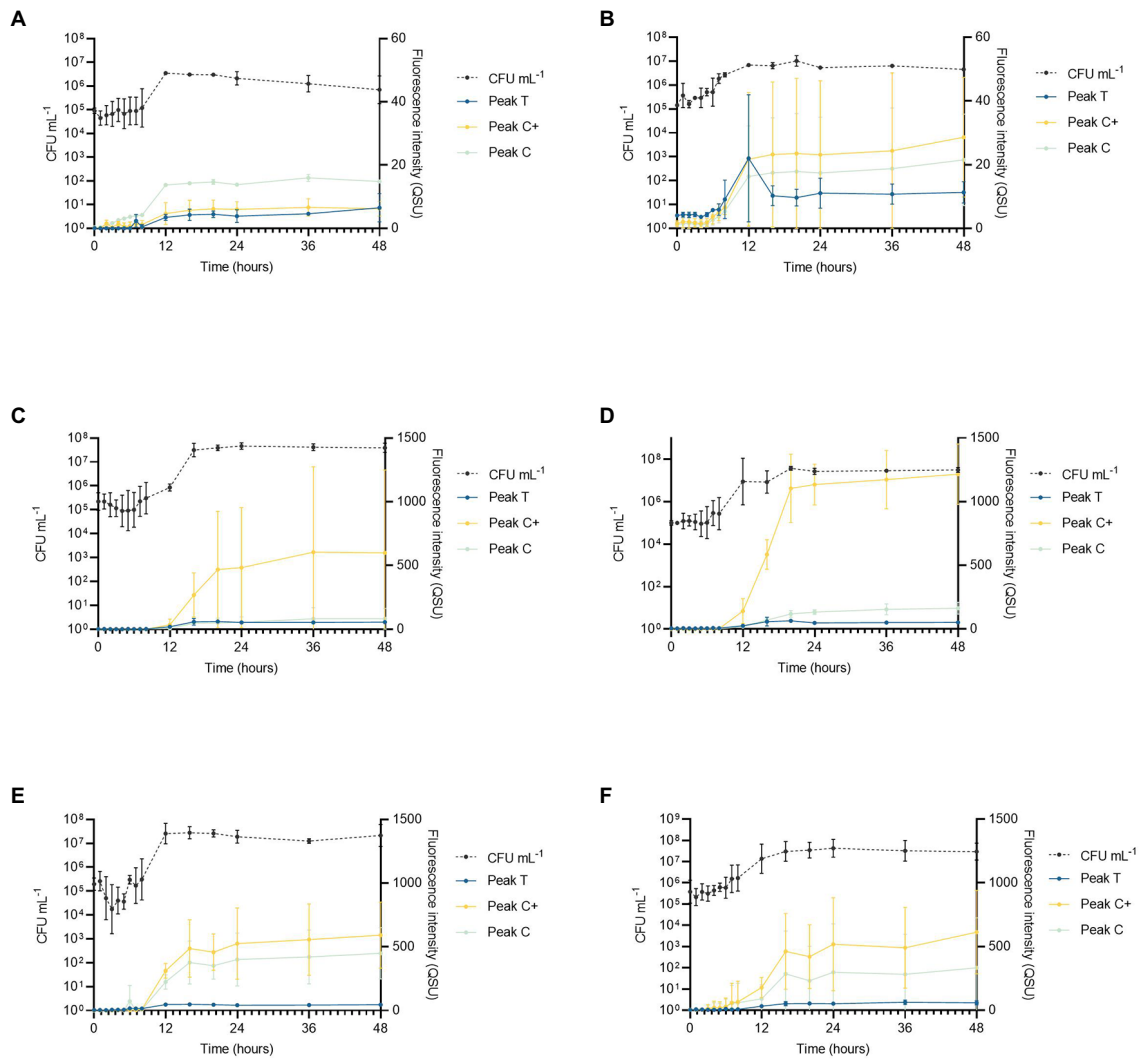
*Pseudomonas aeruginosa* growth curve data showing cell numbers (CFU ml^−1^) relative to fluorescence intensity over 48 h for Peaks T, C, and C+ for the following experimental conditions (*n* = 3 biological and technical replicates): **(A)** low nutrient, no DOC (SFW0), **(B)** high nutrient, no DOC (SFW1), **(C)** low nutrient, limited DOC (SFW2), **(D)** high nutrient, limited DOC (SFW3), **(E)** low nutrient, excess DOC (SFW4), and **(F)** high nutrient, excess DOC (SFW5).

By using glucose as the sole carbon source, it was possible to investigate the bacterial production of AFOM over a 48 h period. At time zero, all inoculated SFW cultures under all conditions (SFW0-5, see Table 2) exhibited minimal fluorescence properties (< 5 QSU). Complex fluorescence signatures were produced under all six experimental conditions (SFW0–5), where the dominant fluorescence Peaks T, C, and C+ were present at the end of the 48 h incubation period (see Figure 1; Table 3). Notably, the production of these AFOM peaks was observed within both high and low nutrient conditions (SFW0 and SFW1) containing no glucose-DOC, albeit at low fluorescence intensities (< 50 QSU) as shown in Figures 1A, 2A and Table 4.

**Table 3:** Fluorescence intensities at 48 h for all peaks (T, C, and C+) under all conditions (SFW0–5).

	No DOC		5 mgL^−1^ DOC		800 mgL^−1^ DOC	
	Low nutrient	High nutrient	Low nutrient	High nutrient	Low nutrient	High nutrient
	QSU (48 h)	QSU (48 h)	QSU (48 h)	QSU (48 h)	QSU (48 h)	QSU (48 h)
T	6.5 (±3.7)	11.3 (±2.4)	56 (±4.1)	51.8 (±1.1)	45.5 (±1.3)	58 (±14.6)
C	14.8 (±0.13)	21.5 (±10.2)	81.5 (±66.4)	162.7 (±38)	449 (±164)	332.6 (±364)
C+	6.2 (±1.9)	28.6 (±13.3)	599.7 (±530.3)	1215.8 (±193.8)	592 (±212.2)	612.7 (±303.3)

Fluorescence intensities are reported in quinine sulfate units (QSU). Variation is also provided in the form of SD (denoted by ±).

**Table 4:** Fluorescence intensities showing total fluorescence (*f*_total_) normalized per 10^6^ CFU.

	No DOC				5 mgL^−1^ DOC				800 mgL^−1^ DOC			
	Low nutrient		High nutrient		Low nutrient		High nutrient		Low nutrient		High nutrient	
	*f_QSU/CFU_*	%	*f_QSU/CFU_*	%	*f_QSU/CFU_*	%	*f_QSU/CFU_*	%	*f_QSU/CFU_*	%	*f_QSU/CFU_*	%
T	50.2 (±34.4)	8	152.6 (±14.7)	49	391.8 (±507.5)	40	647.7 (±480.1)	47	1346.3 (±170.8)	51	229.7 (±211.3)	10
C	128.6 (±52.5)	20	66.8 (±37.8)	22	346.5 (±402.0)	36	251.8 (±145.2)	19	693.2 (±22.7)	26	929.6 (±2052.8)	42
C+	462.2 (±261.2)	72	88.1 (±48.5)	28	237.0 (±212.0)	24	465.9 (±271.7)	34	623.2 (±313.9)	23	1069.3 (±2300.6)	48
*f_total_*	641.1		307.4		975.3		1365.3		2662.8		2228.7	

The *f*_total_ is defined as the sum of Peaks T, C, and C+ at each time point, with all time points over the experimental duration added together for each experimental condition, and is reported in *f*_QSU/CFU_. The relative contribution of individual peaks to the *f*_total_ is also shown in the form of a percentage value.

Viable bacterial cell counts of extracted samples show that the exponential phase of bacterial growth consistently occurred between 6 and 12 h under all experimental conditions as shown in Figure 2. This corresponds to observed increases in the intensities of all AFOM peaks under all conditions (SFW0–5). In all conditions, except SFW2 (low nutrients, limited DOC), bacterial cell replication continued until 16 h as can be deduced from Figure 2C. Upon inoculation of all samples, at 0 h, no significant difference was observed in viable counts (CFU ml^−1^) providing confidence that inoculum density was stable between the different experimental conditions. Between 0 and 6 h, a lag phase was observed during which viable cell counts remained within the range of 5 Log_10_ CFU ml^−1^ for all conditions. However, during exponential growth, cell numbers increased by between 1 and 2 Log, ranging between 1–7 × 10^5^ CFU ml^−1^ at the beginning of the growth phase, and 5 × 10^6^–5 × 10^7^ CFU ml^−1^ at the end of the growth phase. During this time, the growth rate of the bacterial communities can be seen to diverge as a function of nutrient and DOC conditions. Within high nutrient, excess carbon conditions, (SFW5) significantly higher CFU ml^−1^ values (6.7 × 10^5^ CFU ml^−1^) were observed after 5 h (*p* < 0.01) when compared to all other experimental conditions (SFW0–4). From 8 h of incubation, both the low and high nutrient conditions with no DOC (SFW0–1) had significantly lower cell counts (*p* < 0.05) than all other conditions (SFW2–5). In addition, significant differences in cell numbers were observed between limited DOC (SFW2–3) and excess DOC (SFW4–5) conditions (*p* < 0.05); while differences in cell number were found to be significantly affected by the provision of DOC in the system, the availability of nitrate and phosphate was not found to result in significantly higher cell numbers (*p* > 0.05), suggesting that the community is not limited by nitrate or phosphate within this experimental system.

The fluorescence intensities of all peaks under all conditions (SFW0–5) vary over the experimental time period of 48 h. The fluorescence peaks mimic the lag phase for 0–6 h before increasing in concert with the exponential growth phase of the *P. aeruginosa* community studied. Peak T fluorescence exhibited the largest increase in intensity between 6 and 16 h. For low nutrient conditions with 0, limited and excess DOC (SFW0, SFW2, and SFW4), increases of 778% for Peak T, 65% for Peak C, and 22,382% for Peak C+ were observed. For high nutrient conditions, under the same DOC conditions (SFW 1, SFW3, and SFW5) increases of 756% for Peak T, 216% for Peak C, and 682% for Peak C+ were observed. After 16 h, the observed fluorescence intensities plateaued after increases of 52, 10, and 2% for conditions with low nutrients and excess DOC conditions (SFW0, 2, and 4) for Peaks T, C, and C+. For high nutrient conditions (SFW1, 3, and 5), the observed increases were 12, 0, and 20% for Peaks T, C, and C+, respectively.

In a time-series analysis of all fluorescence peaks over the entire experimental period (0–48 h), it was found that there was a significant change in fluorescence intensity over time for the majority of peaks investigated across all of the experimental conditions. For low nutrient, no DOC conditions (SFW0), Peaks T and C were found to significantly change over the experimental duration (*p* < 0.01). However, Peak C+ was not seen to significantly change over the experiment (*p* > 0.05). For high nutrient, no DOC conditions (SFW1), no peaks were found to change significantly over the experimental period (*p* > 0.05). For low nutrient conditions with limited DOC (SFW2), Peaks T and C were found to change significantly (*p* < 0.05); however, Peak C+ was not (*p* > 0.05). All other conditions (SFW3-5) were found to display significant changes over the experimental duration (*p* < 0.05).

The relationship between individual fluorescence peaks varies significantly throughout the experimental duration. Under high nutrient conditions and zero DOC (SFW1), low nutrient conditions and limited DOC (SFW2), and high nutrient conditions and excess DOC (SFW5), there are no significant differences (*p* > 0.05) observed between Peaks C and C+, suggesting that the production of this AFOM is interrelated. In contrast, for low nutrient, zero DOC (SFW0), Peak C fluorescence intensity is seen to decrease by 1.2 QSU (± 0.8) over the final 24 h (late stationary phase), while Peak C+ decreases by only 0.4 QSU (± 0.3), resulting in a divergence between these two peaks at the end of the experimental period. This was found to be statistically significant (*p* < 0.05). For high nutrient conditions, limited DOC (SFW3), the relationship between Peaks C and C+ is statistically different, where the greatest increase in Peak C+ is observed between 36 and 48 h, increasing by 42.3 QSU (± 9.4) while Peak C increases by 7.8 QSU (± 5.3; *p* < 0.05). For all other conditions (SFW3 and SFW4), there remains no significant difference between these peaks over the experimental duration. The relationship between Peak T and C/C+ also varies, with only low nutrient, zero DOC (SFW0) displaying a significant difference between Peaks T and C throughout the experimental period (*p* < 0.05) for all but the first 2 h. High nutrient, limited DOC (SFW3), display the most significant differences between all peaks, where the difference is over 1,000 QSU between Peak C+ and Peaks T and C (*p* < 0.05; Figure 2). For Peaks T and C+, this significant difference is present throughout the experimental period (*p* < 0.05). For high nutrient, excess DOC (SFW5), the variation observed between the three biological replicates was found to be substantially higher than was seen under all other conditions. For Peaks C and C+, there was found to be a mean of 332.6 and 612.7 QSU, and a SD of 364 and 303.3, respectively, at 48 h. Comparatively, there is less variability between biological replicates for low nutrient, no DOC conditions (SFW0), with means of 14.8 QSU for Peak C and 6.2 QSU for Peak C+, and SDs of 0.1 for Peak C and 1.9 for Peak C+. Observed variability was determined to be a result of inherent biological variation which is discussed further in “Discussion.” Lower SD was observed in conditions that comprised of lower nutrient conditions.

### Effects of DOC and Nutrients (NO_3_^−^ and PO_4_^3−^) on AFOM Production and Processing

**Figure 3:**
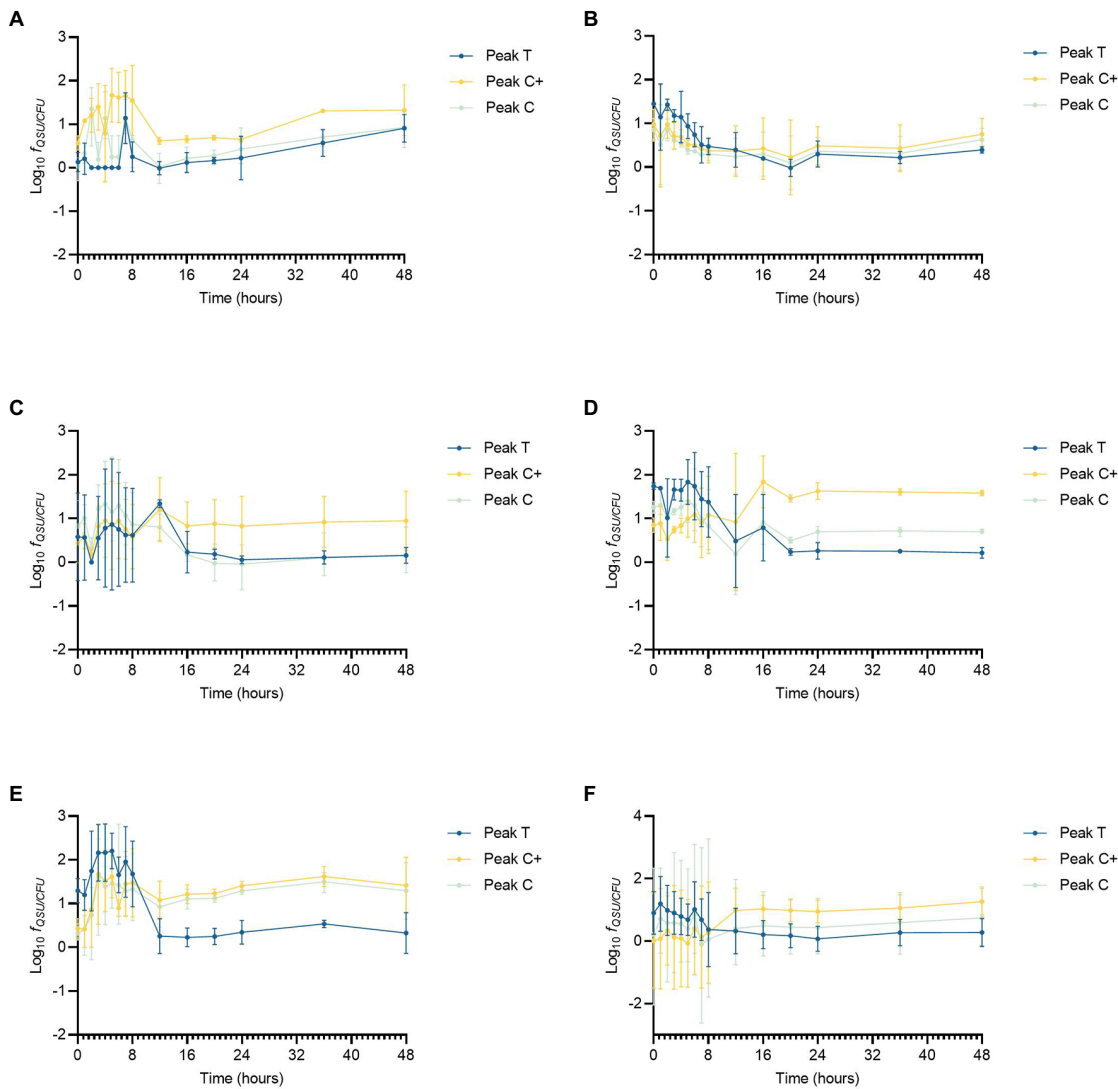
Peaks T, C, and C+ fluorescence data corrected for enumeration, displaying Log_10_ QSU per 10^6^ CFU (*f_QSU/CFU_*) for *Pseudomonas aeruginosa* growth curve data under the following experimental conditions (*n* = 3 biological and technical replicates): **(A)** low nutrient, no DOC (SFW0), **(B)** high nutrient, no DOC (SFW1), **(C)** low nutrient, limited DOC (SFW2), **(D)** high nutrient, limited DOC (SFW3), **(E)** low nutrient, excess DOC (SFW4), and **(F)** high nutrient, excess DOC (SFW5).

To compare directly the effect of contrasting nutrient regimes on AFOM processing, QSU fluorescence data were normalized to bacterial enumeration to account for the changing density of viable cells during the growth curve period (Figure 3). For this, QSU data were enumeration-corrected to every 10^6^ CFU and the data logged for all conditions for comparison (expressed as *f_QSU/CFU_*). For the experimental time period, the relationship between viable cell numbers and Peaks T, C, and C+ fluorescence is not linear for any of the conditions studied (Figure 3). Large fluctuations occurring in per-cell fluorescence (*f*_QSU/CFU_) over the duration of the experiment were observed. Under all experimental conditions, the period of greatest variation in *f*_QSU/CFU_ occurs between 0 and 12 h. An increase in per cell fluorescence can initially be seen under all conditions, with maximum fluorescence intensities being reached during the first 8 h of the experiment. This coincides with the beginning of the bacterial exponential growth phase as seen in Figure 2, which occurs between 6 and 12 h, during which the bacterial growth rate is at its highest. In high nutrient conditions with limited DOC (SFW 3), Peak C+ reaches its maximum *f*_QSU/CFU_ at 16 h, and in high nutrient conditions with excess DOC (SFW5), Peak C+ reaches its maximum *f*_QSU/CFU_ at 12 h. A sharp decrease in *f*_QSU/CFU_ is then observed until 12 h in conditions containing zero and excess DOC (SFW0, SFW1, SFW4, and SFW5, Figures 3A,B,E,F) for all peaks except for Peak C in SFW5. Under limited carbon conditions (SFW2 and SFW3, Figures 3C,D), *f*_QSU/CFU_ continues to decrease until 16 and 24 h for low and high nutrient conditions, respectively. While the *f*_QSU/CFU_ remains low for the remaining duration of the experimental period, all conditions (with the exception of SFW3), experience a gradual increase during the final 24 h of the experiment coinciding with the late stationary phase of the bacterial community.

*f*_QSU/CFU_ is influenced by the availability of nutrients and DOC in the system. Table 4 shows the influence of the experimental conditions on the ratio of Peaks T, C, and C+. The relative contribution of each fluorescence peak to the total fluorescence observed as a function of nutrients and DOC is shown. In conditions with zero and limited DOC, higher nutrient availability was found to result in more Peak T production, with a higher ratio of Peak T relative to Peaks C and C+. Under excess DOC conditions, however, increased nutrient availability was found to reduce the production of Peak T relative to Peaks C and C+, with the latter peaks accounting for a larger proportion of the total fluorescence. For experimental conditions containing zero DOC, the maximum *f*_QSU/CFU_ for Peak T in high nutrient conditions was substantially higher (102.3, ±20.5) in comparison to low nutrient conditions. When DOC is limited, the *f*_QSU/CFU_ for Peak T was elevated by 255.9 (±45.7) in low nutrient conditions compared to higher nutrient conditions. Interestingly, for conditions containing excess DOC, the *f*_QSU/CFU_ for Peak T associated with high nutrients was significantly less (1116.6 ± 257.8) when compared to low nutrient conditions (*p* < 0.01). For Peaks C and C+, conditions with no DOC show that the addition of nutrients results in a decrease in fluorescence intensity of 61.8 (± 36.7) *f_QSU/CFU_* for Peak C and 374.2 (±196.6) *f_QSU/CFU_* for Peak C+. However, for high nutrients and excess DOC conditions, higher fluorescence intensities for Peak C are observed in the high nutrient conditions. This equates to an additional *f*_QSU/CFU_ of 228.8 (±168.2).

## Discussion

### AFOM Processing Over 48 h

The data presented here demonstrate that under all conditions, *P. aeruginosa* is capable of producing a complex range of AFOM including Peaks T, C, and C+, in a SFW model system. This includes the production of materials that fluoresce in both the autochthonous (protein-like) and allochthonous (humic-like) optical regions. Furthermore, material that has conventionally been assigned as autochthonous and allochthonous have been produced within this biological model in low and available DOC conditions. The production of this material relative to the bacterial cell numbers in the system was shown to vary over time, commensurate with the stage of biological growth occurring in the community.

It has been previously shown that bacterial communities are capable of producing semi-labile humic-like material from *in situ* incubation experiments within marine systems (Koch et al., 2014), consistent with the theory of the microbial carbon pump (Jiao et al., 2011). This has been characterized, using molecular techniques such as ultra-high-resolution mass spectrometry, as consisting of higher molecular-weight material generated from biologically labile substrates such as glucose (Koch et al., 2014). More recently, model freshwater studies have shown the production of optically active compounds that are fluorescent in the high molecular-weight, humic-like region using high-carbon, and high-nutrient microbiological growth media (Fox et al., 2017, 2018). In support of this study, the data presented here clearly demonstrate that *P. aeruginosa* is capable of producing AFOM peaks commonly considered to be allochthonous in nature, from a low-nutrient base media containing no additional nitrate, phosphate, or DOC. This suggests that while the capacity of bacteria to produce complex AFOM *in situ* is enhanced by the availability of a labile, low molecular weight carbon source (glucose), and an abundance of nutrients, they are also capable of utilizing very minimal concentrations of essential nutrients to demineralize and/or utilize carbon derived from the microbial community to produce fluorescing material. It is notable that conditions containing no introduced glucose-DOC still produced substantial fluorescence signatures over the experimental period (that is, 11.3, 21.5, and 28.6 QSU for Peaks T, C, and C+ in high nutrient, no DOC conditions at 48 h) which, while lower than the 58, 332.6 and 612.7 QSU for Peaks T, C, and C+ seen in high nutrient, excess DOC conditions, still represents a significant increase in fluorescence over the experimental period. It is likely that this occurred as a result of DOC delivered into the system from the microbial inoculum in the form of bacterial biomass which was utilized by the community as an organic substrate to generate fluorescing compounds. The introduction of bioavailable DOC from the microbial community may have occurred as a result of a number of factors; for example, cell death and subsequent cell lysis, or the export of compounds such as iron-scavenging siderophores into the system.

Data presented in Figure 3 clearly demonstrate that the relationship between AFOM peaks and cell density is not correlated for any conditions within this model system. The intensity of the AFOM produced per bacterial cell varies both throughout the experimental period and between different conditions of nutrients and carbon. By observing these dynamics over time (at hourly intervals between 0 and 8 h) variations in *f*_QSU/CFU_ are observed between all experimental conditions studied. Peak T was observed in all conditions and always displays an increase in fluorescence intensity between 8 and 16 h. This suggests that while DOC and nutrient availability heavily influence the maximum intensity of the fluorescence peaks produced, ultimately it does not affect the inevitable pattern of AFOM production over time. There are large fluctuations observed in *f*_QSU/CFU_ during the first 12 h period of all experiments, with Peak T reaching its maximum *f*_QSU/CFU_ between 0 and 7 h in all conditions, before sharply decreasing thereafter. The sharp increase in maximum *f*_QSU/CFU_ during the first several hours of the experiment suggests that an increase in Peak T fluorescence intensity may represent a precursor to the start of the exponential growth phase, indicating the upregulated metabolic state the bacterial community is undergoing prior to cell replication. This variable relationship between *f*_QSU/CFU_ of Peak T, and the increase seen in the *f*_QSU/CFU_ prior to a period of rapid cell multiplication, strongly supports the use of Peak T as a marker for an active bacterial population under model conditions, as has been evidenced in recent literature (Fox et al., 2017, 2018). As such, this model highlights the complexity of the relationship between bacterial activity and observed fluorescence, underscoring the fact that while Peak T presents a useful tool for monitoring microbial presence in surface waters, there may be challenges associated with using this technique as a direct enumerator for bacterial cells as has been previously reported (Sorensen et al., 2015, 2018), particularly within dynamic systems which are heavily influenced by nutrient influxes. It should be noted that the viable count method used to enumerate bacterial cells in this study does not account for every cell present within the system, for example viable but non-culturable cells. Therefore, while methodological consistency ensures relative accuracy within the study, some of the cells present within that are not represented within the bacterial cell number data, may still be contributing to the observed fluorescence signature.

Peak C/C+ fluorescence also exhibits a non-linear relationship with bacterial cell density over the duration of the experimental period. Following the initial fluctuation in *f*_QSU/CFU_ between 0 and 12–16 h, the *f_SU/CFU_* can be seen to increase, gradually, for the duration of the experiment for all conditions except for high nutrient and limited DOC. This could possibly be caused by the persistence of compounds that comprise Peak C/C+ FOM within the system (i.e., are not acted upon by the bacterial population). This is supported by previous literature which demonstrates the microbial production of less labile, higher molecular weight material (known to fluoresce in the Peak C/C+ region) albeit in the marine environment, from a simple glucose carbon substrate, which exhibited persistence for up to 2 years (Kinsey et al., 2018). Our data shows the *in situ* production of Peaks C and C+ within a freshwater matrix, and their subsequent accumulation and persistence within the system throughout the experimental duration of 48 h. Material that fluoresces in this region has conventionally been associated with allochthonous material that is transported into surface waters from the surrounding catchment, rather than as a result of the direct *in situ* production by microorganisms. While the *in situ* production of this material has previously been seen in marine environments, to our knowledge, our study is the first to show this phenomenon in a controlled laboratory environment using a representative SFW matrix. Further work is required to determine the relative recalcitrance of the presumed higher molecular weight FOM that is produced, by monitoring the fluorescence in this region over longer periods of time and in the presence of a more diverse microbial community. Recent literature has suggested that the origin of Peak C+ is partly derived from extracellular microbial products (Sorensen et al., 2020; Fox et al., 2021) However, to date, the overwhelming consensus is that Peaks C and C+ in freshwater environments are associated with material that is allochthonous in origin (Coble et al., 2014).

### Effects of DOC and Nutrients (NO_3_^−^ and PO_4_^3−^) on AFOM Production and Processing

The concentration of DOC, nitrate, and phosphate was found to have an impact on the production and processing of AFOM by the bacterial community within the SFW models studied here. The data presented in Table 4 show that higher concentrations of DOC resulted in higher maximum *f*_CFU/QSU_ values (sum of Peaks T, C, and C+). For all low nutrient conditions, the sum of the maximum *f*_QSU/CFU_ is higher with increasing DOC conditions. Similarly, for high nutrient conditions, incremental increases of the maximum *f*_CFU/QSU_ are observed. This shows that with higher DOC availability, bacteria are capable of producing increased quantities of AFOM per bacterial cell. This suggests that carbon availability represents a limiting factor to AFOM production within the model system studied. This further supports the notion that AFOM observed in the natural environment will be a direct consequence of the activity of the bacterial cells present and the quantity and availability of a carbon substrate from which to produce AFOM.

While increased DOC availability results in overall increases to the maximum *f*_QSU/CFU_, there are substantial differences in the relative contribution of each of the studied peaks to the *f_total_*. Moreover, the DOC concentration appears to have an influence on the effect of nutrient additions on AFOM production. In experimental conditions containing no added DOC and 5 mg L^−1^ DOC, the addition of nutrients results in an increase in Peak T fluorescence and a decrease in Peaks C and C+. However, in high nutrient and excess DOC conditions, there is a substantial decrease in Peak T fluorescence, and an increase in the production of Peaks C and C+, in comparison to the observed fluorescence exhibited in low nutrient, excess DOC conditions. This suggests that when the availability of carbon substrate in the system is a limiting factor, the production of Peak T takes precedent within the system. Peak T is considered to represent intracellular structural and functional compounds such as amino acids, which are essential for cell growth and replication (Coble, 2007) and therefore may take priority when carbon is limited, or in high demand, to the bacteria within the system. However, when carbon is available in excess, additional nutrient resources may be diverted to the production of higher molecular weight compounds such as Peaks C and C+. This may suggest that the community is producing a pool of more recalcitrant compounds to store carbon while DOC is available in excess. Conversely, when DOC is available in excess but nutrient resources are limited, the ability to produce these higher molecular-weight compounds may be compromised, leading to an excess production of smaller, simpler proteinaceous material. By providing a nutrient-limited baseline which can be incrementally manipulated to study the effects of controlled nitrate, phosphate, and DOC loading on the system, we have been able to demonstrate that the availability of these nutrients has a significant impact on the fluorescence production by the bacterial species present within our controlled model system. This further supports previous findings that fluorescence intensity measurements cannot be used as a surrogate measure of bacterial enumeration. When monitoring fluorescence within natural systems, it may be important to consider the effect of changing environmental conditions such as nutrient and DOC availability on the intensity and ratios of fluorescence peaks present to obtain an insight into the functioning of a system.

The DOC availability within the system is also seen to influence the normalized fluorescence intensity per bacterial cell (*f_QSU/CFU_*). Greater variations were observed in high-nutrient, excess DOC conditions than in any other experimental condition (Figure 2) in relation to Peaks C and C+. This is possibly because organic matter produced by *P. aeruginosa* is known to fluoresce in the Peak C+ region, which also corresponds to the observed fluorescence properties of the siderophore pyoverdine (Wasserman, 1965). Pyoverdine is an extracellular iron-scavenging metabolite, and is strongly pigmented (Meyer, 2000). It is possible that the rapid production of Peak C+ shown in our data can be attributed to the production and exporting of pyoverdine from the bacterial cells. The production of pigmented, water-soluble extracellular siderophores by *Pseudomonas* species has long been known to be influenced by a range of environmental factors (Cornelis et al., 2002). The differential expression of these fluorescent pigments as a result of external factors such as nutrient availability could suggest that under high-nutrient conditions, external factors controlling the rate of fluorescence production may be affecting the system, resulting in the variability seen only within the high-nutrient, excess DOC conditions. The variability in the expression of fluorescent pigments by bacteria as a result of nutrient and DOC availability further highlights the complexities associated with using fluorescence intensity as a predictor for bacterial enumeration, but could support its application as a useful biomarker for a metabolically active microbial population, particularly in relation to its responses to changing environmental conditions in natural waters. The observed high variations in Peak C/C+ fluorescence intensities within the high nutrient, excess DOC conditions could be attributed to metabolic differences between independent biological populations of the same species. Observed variations between individual fluorescence measurements within experimental repeats are much lower, eliminating instrumental variability. Therefore, we postulate that the variations in exported humic-like AFOM are driven by metabolic variations between discrete populations of *P. aeruginosa*. This is further supported by the *f_QSU/CFU_* data which demonstrates that the measured fluorescence values are independent of cell numbers. In addition, while pyoverdine fluoresces in the humic-like region, it is not considered to represent humic-like material. Siderophores are usually considered a group of low molecular weight compounds, often between 500 and 1,500 Daltons (Da; Hider and Kong, 2010). Pyoverdine has a molecular weight of 1335.4 Da which, while still considered to be low molecular weight, does represent a higher molecular weight than glucose, at 180 Da. This suggests the possible production of higher molecular weight compounds (pyoverdine) from a simple and labile carbon source (glucose) is occurring.

In the future, further investigation into the relationship between microbial metabolism and AFOM production would be required to help develop our understanding, including an investigation of a broader range of species to obtain a more representative bacterial community inoculum. In addition, changes could be made toward the development of a more environmentally representative model. This may include investigating bacterial AFOM production under ambient environmental temperature conditions, or employing a range of environmentally representative nutrient and DOC conditions. It would also be of merit to investigate the continued processing of AFOM over a longer experimental period (i.e., weeks) to determine the relative recalcitrance of the material produced. A limitation of this study is the lack of data pertaining to the levels of nitrate, phosphate and DOC within the system throughout the experimental time period. Future work will incorporate these measurements to further delineate the relationship between nutrient availability and AFOM production. Furthermore, the inclusion of a mixed-community model encompassing planktonic bacteria along with biofilm and algal communities would be more relevant to systems observed in the natural environment. In addition, while the relationship between AFOM production and bacterial metabolism is postulated in this study, further work would be required to verify this experimentally. This may include the addition of direct measurements of metabolic rate or bacterial production alongside fluorescence and enumeration measurements to determine the relationship.

## Conclusion

- *Pseudomonas aeruginosa* is capable of producing a range of AFOM including Peaks T, C, and C+ from a simple carbon source within a SFW model system. This includes the production of material which fluoresces in the allochthonous region, which is conventionally associated with higher molecular weight material within freshwater systems.
- The relationship between fluorescence intensity and bacterial cell number is nonlinear over the experimental time period. During times of known upregulated metabolism, e.g., the exponential bacterial growth phase, higher *f*_QSU/CFU_ is observed, supporting the use of fluorescence as a marker for upregulated microbial activity rather than enumeration.
- Both fluorescence intensity and fluorescence peak ratios are influenced by the concentration of nitrate, phosphate, and DOC within a SFW model system. While higher DOC concentrations result in higher total fluorescence, this also influences the response of the bacterial community to the introduction of nutrients.

## Data Availability Statement

The original contributions presented in the study are included in the article/supplementary material, further inquiries can be directed to the corresponding author.

## Author Contributions

EP, DR, and RT: conceptualization and methodology. EP: data curation, investigation, and writing—original manuscript draft. EP, DR, RT, and SS: formal analysis. DR, RT, and JA: funding acquisition. EP and DR: project administration. DR, RT, SS, and JA: supervision. SS, RT, and DR: manuscript writing, reviewing, and editing. JA: manuscript review only. All authors contributed to the article and approved the submitted version.

## Funding

This study received partial funding from Chelsea Technologies Ltd., who were not involved in the study design, collection, analysis, interpretation of data, or the writing of this article (manuscript reviewing only) nor the decision to submit it for publication. Other funding was provided by the University of the West of England, Bristol (PhD funding) and the Natural Environment Research Council, UKRI as part of delivering grant NE/R003106/1.

## Conflict of Interest

JA is employed by Chelsea Technologies Ltd. who provided partial funding for this study. The funder Chelsea Technologies Ltd. had the following involvement with the study—reviewing of final manuscript only.

The remaining authors declare that the research was conducted in the absence of any commercial or financial relationships that could be construed as a potential conflict of interest.

## Publisher’s Note

All claims expressed in this article are solely those of the authors and do not necessarily represent those of their affiliated organizations, or those of the publisher, the editors and the reviewers. Any product that may be evaluated in this article, or claim that may be made by its manufacturer, is not guaranteed or endorsed by the publisher.
